# Supplementary material for: Impacts of resistance training combined with vibration training on the IGF-1/PI3K/AKT/FOXO3 axis and clinical outcomes in patients with sarcopenia: A protocol for a randomized controlled trial
Source: PLoS One. 2025 Sep 26;20(9):e0333343. doi: 10.1371/journal.pone.0333343 (PMC12468885; doi:10.1371/journal.pone.0333343)
Supplement: S2 File — (PDF) [file pone.0333343.s002.pdf]

成 都 体 育 学 院

伦理委员会审批证明

成体伦理〔2024〕66 号

我校研究人员 周昊阳 等开展的科学研究: 抗阻训练结合振动训练对老年肌少症患者 IGF-1/PI3K/AKT/FOXO3 信号通路的影响 经审查，在研究过程中涉及人的生物医学研究和相关技术应用，包括化学和生物学方法在人体上对人的生理、病理现象以及疾病的诊断、治疗和预防方法进行研究的活动；研究形成的医疗卫生技术或者产品在人体上进行试验性应用的活动，符合我国现行的相关伦理研究的相关政策法规规定，现予以批准实施。

特此证明。

成都体育学院伦理委员会

2024 年 4 月 23 日

Chengdu Sport University

Certificate of approval by the ethics committee

ChengTi LunLi〔2024〕66

The scientific research conducted by our researcher **Haoyang Zhou**: **Effects of resistance training combined with vibration training on the IGF-1/PI3K/AKT/FOXO3 axis in a population with sarcopenia.** have been reviewed, and the research process involves human biomedical research and related technology applications, including chemical and biological methods in the human body to study human physiological and pathological phenomena and disease diagnosis, treatment and prevention methods: research formed medical and health technology or products experimental application activities, In line with the relevant policies and regulations of ethical research in China, it is hereby approved for implementation.

It is hereby certified.

Ethics Committee of Chengdu Sport University

23/4/2024

Mingjian Wang

23/4/2024
