# Supplementary material for: Impacts of resistance training combined with vibration training on the IGF-1/PI3K/AKT/FOXO3 axis and clinical outcomes in patients with sarcopenia: A protocol for a randomized controlled trial
Source: PLoS One. 2025 Sep 26;20(9):e0333343. doi: 10.1371/journal.pone.0333343 (PMC12468885; doi:10.1371/journal.pone.0333343)
Supplement: S3 File — (DOCX) [file pone.0333343.s003.docx]

1.诚信申明：

郑重声明：本人所注册的《抗阻训练结合振动训练对老年肌少症患者IGF-1/PI3K/AKT/FOXO3信号通路的影响》，是周昊阳等人在廖远朋的指导下，合作进行的研究工作。除文中已经注明引用的内容外，本论文不包含任何其他个人或集体已经发表或撰写过的作品成果。我们将保证操作严格按照试验规程和数据记录的真实性。本研究参与者无任何利益冲突，对做出重要贡献的个人和集体，将在在文中以明确方式标明并致谢。本人完全意识到本声明的法律结果由本人承担。

2.研究题目：

抗阻训练结合振动训练对老年肌少症患者IGF-1/PI3K/AKT/FOXO3信号通路的影响

3.研究计划书版本号：

V1.0

4.经费来源：

学院资助加自费

5.研究背景：

肌少症是一种与衰老相关的进行性疾病，以骨骼肌质量降低、肌肉力量下降和/或身体活动能力下降为特征。根据亚洲肌少症工作组(AWGS)指南，中国≥60岁老年人肌少症患病率为9.3%，其中男性为6.4%，女性为11.5%。意外跌倒、上下肢骨折、肢体残疾、认知障碍、生活质量下降甚至死亡等不良结局与肌少症的进展相关。老年肌少症患者的变化不仅体现在临床结局和生理结局上，如年龄相关性炎症、代谢异常、内分泌改变等。

在老年人(≥60岁)中，肌肉减少症与IGF-1降低所表明的合成代谢激素水平降低有关。叉头框蛋白O3 (Forkhead-Box Protein O3, FOXO3)是IGF-1信号通路的下游成员，在肌肉骨骼系统的蛋白质降解和合成/分解代谢蛋白质稳态中起主导作用。通过PI3K/AKT通路的调节，胰岛素样生长因子1 (IGF-1)的低水平导致FOXO3的高活性。产生的靶基因是导致骨骼肌萎缩的泛素连接酶Atrogin-1。在肌肉骨骼系统的许多控制基因中，FOXO3是关键的，因为它在肌肉生长过程中失活。因此，FOXO3的失活可能是治疗肌肉萎缩的潜在靶点。因此，应采取一些措施预防和(或)延缓肌少症的发展。

由于缺乏特别有效的药物来治疗肌少症，目前的研究表明，非药物治疗，尤其是运动训练，仍然是最有希望的治疗方式，可以显著改善老年肌少症患者的肌肉功能和身体健康。目前，抗阻训练(RT)是老年肌少症患者最常用的运动训练之一。但RT对老年人技术要求高，容易产生肌肉疲劳。这些可能会降低老年人的依从性，尤其是虚弱的老年人，并且很难在临床环境中普遍实施。振动训练(VT)是一种利用不同振动频率和振幅的振动平台在不同体位进行训练的方法，是一种改善老年人肌少症的前瞻性策略。近年来，VT的应用越来越广泛地用于延缓老年肌少症患者的肌肉功能丧失。meta分析结果显示，VT可以改变肌肉质量、肌肉力量和体能的下降趋势;同时，多项研究证实VT对肌少症有积极作用。因此，VT联合RT可能是一种潜在的改善肌少症的替代训练方法。

6.研究目的：

探讨阻力训练结合振动训练对肌少症患者IGF-1/PI3K/AKT/FOXO3轴的影响。并通过单盲随机对照试验，评估两种运动联合治疗策略在分子水平上对肌少症患者的生理变化是否有显著影响。7.纳入和排除标准：

纳入标准：

纳入标准为年龄介于65至80岁，并根据AWGS 2019标准诊断为肌肉减少症的老年人，同时无严重疾病，如未控制的高血压、高脂血症、高血糖症和糖尿病，及没有未经治疗的疾病，如胆结石或肾结石，以及传染病，参与者没有固定的运动习惯超过3个月。

排除标准：

1) 未控制的高血压、高脂血症或糖尿病；2) 未治疗的胆结石、肾结石或感染性疾病；3) 病理性骨病或慢性炎症性疾病（如类风湿性关节炎）； 4) 任何可能影响完成研究中所需锻炼能力的疾病；5) 需要安装起搏器的心血管疾病或恶性肿瘤；6) 急性骨折或严重骨关节炎；7) 目前正在使用已知会显著影响肌肉代谢或激素平衡的药物，包括但不限于全身性皮质类固醇、合成代谢类固醇或激素替代疗法。此外，所有参与者当前用于心血管或代谢性疾病的药物（如β受体阻滞剂、他汀类药物、胰岛素增敏剂）将在基线时记录。若发现组间存在不平衡，这些变量将在统计分析中作为协变量考虑；8) 当前或近期（过去3个月内）使用可能影响肌肉代谢或肌少症进展的营养补充剂，如蛋白粉、氨基酸、维生素D或钙补充剂。

8.设计方案，设计模式图，附SPIRIT模版

平行设计随机对照实验

9.样本量估算：

根据之前的相关研究，使用GPower, V.3.1.9.7软件计算样本量为至少73人，alpha为0.05,power (1 - β)为0.80，并考虑20%的流失率。因此，我们将招募96名老年人，每组24人。

10.随机和隐蔽分组的方法：

对自愿参加研究的成年老年人进行资格筛选，然后随机平均分配到 RT、VT、RVT 或对照组。将使用 Excel 软件根据 96 名参与者的报名时间对其进行顺序编码，然后使用公式“=RAND() ”生成相应的随机序列。为方便随机化过程，将对每位参与者生成的随机数进行排序，并应用模运算“=MOD()”，根据余数将每位参与者分配到四组中的一组——0 为 RT 组，1 为 VT 组，2 为 RVT 组，3 为对照组。通过这些步骤生成的随机序列将对 96 名参与者进行排序和分组。随机化工作将由专业信息技术人员执行，以确保研究人员不了解参与者的招募和分组情况，从而保持随机化和盲法过程的完整性。

11.盲法

由于受试者很容易感知出所进行的运动，所以该研究只对对测量人员与评估人员设盲

12.测量指标：

主要指标：握力、步速、IGF-1浓度、PI3K/AKT信号通路、FOXO3 蛋白活性。

次要指标：骨骼肌质量、“起立-行走”计时测试、SF-36健康自测。

13.对参试者有效性认定的定义：

剔除与脱落标准：（1）不符合上述纳入标准而被错误纳入研究者；（2）未按照研究规定的方案进行干预、检查者；（3）受试者在入组后研究开展期间，自行退出本研究，及使用与研究无关或产生严重影响的干预者予以剔除。

14.伦理考量：

已注册，获成都体育学院伦理委员会批准，批件文号为“2024 [66]号”。

15.参试者的征募：

将通过合作社区中心的定期教育讲座、社区中心或社交媒体上的宣传材料以及合作伙伴的推荐来招募受试者。根据AWGS 2019的定义，参与者将通过生物电阻抗分析、握力和步态速度进行筛选。符合虚弱标准的参与者将被邀请参加我们在四川省运动医学重点实验室研究。在签署书面知情同意书之前，所有参与者都将被告知潜在的利益和风险。受试者可以在任何时候无条件退出研究。

16.参试者一般信息的收集：

正式实验前1周，登记受试者姓名、年龄、性别、身高、体重、运动频率、损伤史等信息。

17.基线指标和观测项目：

与测量指标相同。

18.标准操作规程：

（1）实验培训

对RT组、VT组和RVT组进行预培训，使其熟悉相应的培训课程。在预培训后进行正式培训。3个干预组(RT组、VT组和RVT组)在干预前后进行5 min的热身和冷却训练。所有训练课程将由物理治疗师进行。在整个培训期间，研究者将记录参与者的出勤率和依从性。如果受试者因某些原因不能按时参加培训，则将在该周的其他时间进行培训。所有受试者在实验期间不得进行额外的体力活动和/或改变他们的日常生活方式。

（2）抗阻训练组

被分配到RT组的参与者将接受为期12周的锻炼计划，每周进行3次训练。在干预开始前，所有参与者将进行一次最大重复次数测试（1RM），以明确基线肌肉力量并确定个性化训练强度。1RM将通过亚最大负荷测试方法估算，即参与者在选定的TheraBand阻力训练动作（如膝关节伸展、髋关节外展、肘关节屈曲）中，以约60%–80%的自感最大负荷完成3–5次重复。将应用Brzycki公式（估计1RM（kg）＝次最大重量（kg）÷（1.0278−0.0278×最大重复次数））从这些次最大重复次数中估算1RM值。所有测试将在经验丰富的物理治疗师监督下进行，并在组间保持1分钟的标准化休息间隔以减少疲劳。根据估算的1RM值，参与者将被分配适当的TheraBand（按阻力级别颜色编码：黄色、红色、绿色、蓝色、黑色），并指示保持特定的拉伸长度（例如100%或200%）以模拟所需阻力。这允许将负荷调整至约1RM的70%。为提高精准度，将同时使用Borg CR-10量表监测主观用力程度，确保干预期间训练负荷的一致性。整个12周计划的训练强度将设定为1RM的70%。每次训练包含3个阶段：5分钟热身、30分钟主要阻力训练（RT）练习，以及5分钟放松。在5分钟热身阶段，参与者主要进行颈部、肩部、下背部、髋部、膝盖和脚踝的拉伸。参与者将进行每个动作3组，每组10次重复，组间休息1分钟。训练将使用TheraBand进行，以减少自由重量对参与者关节的压力并提高其参与阻力训练的积极性。RT练习将重点针对下肢肌肉（膝关节屈伸、髋关节外展）、上肢肌肉（肩关节伸展/外展、肘关节屈伸）及核心肌肉（包括胸肌推举和胸肌下压）。完成这些练习后，参与者将进行与热身结构相同的5分钟放松。

（3）振动训练组

VT组接受振动训练(VT)，每周3次，每次20分钟，持续12周。全身振动训练(VT)使用产生垂直振动的振动平台(Pro5 AIRdaptive, Power Plate，美国)进行。每次训练持续约20分钟，包括5分钟的热身、10分钟的振动和5分钟的冷却。振动频率逐渐增加至40 Hz，峰峰振幅为4 mm。在VT过程中，受试者将被要求赤脚站在振动平台上，膝盖屈曲60°，如果需要，双手握住振动绳以保持平衡。VT运动应保持1.5分钟，每天重复4组，每组之间休息1分钟。

（4）抗阻训练联合振动训练组

抗阻训练联合振动训练组（RVT group）将接受为期 12 周的抗阻联合振动训练，每周三次。在每次阻力训练结束后，将进行额外的振动训练，振动训练设置参数与 VT 组相同。

（5）对照组

对照组受试者每月在社区中心接受2次60分钟的教育课程。教育课程由特邀专家授课，课程主题为老龄化、健康与肌少症，包括肌少症的定义、发病原因、病理、临床表现及对老年人的不良后果。

19.统计分析方法：

所有数据将使用 IBM SPSS v.20.0 进行分析。将通过双向重复测量方差分析（方差分析；时间点：基线、12、24 周；组别：对照组、RT 组、VT 组、RVT 组）评估主要结果与 Holm-Bonferroni 配对比较。缺失数据将通过多重估算来弥补，协变量（基线 IGF-1、年龄、性别、肌肉质量）将纳入协方差分析模型。效应大小（方差分析采用部分η2；配对比较采用 Cohen's d，置信区间为 95%）将分为小（η2 ≥ 0.01，d ≥ 0.2）、中（η2 ≥ 0.06，d ≥ 0.5）或大（η2 ≥ 0.14，d ≥ 0.8），并附有精确的 p 值。纵向次要结果（如步速）将通过线性混合模型进行分析，并报告各组间的时间交互作用 p 值。探索性亚组分析（p < 0.10）将用于检查性别和基线严重程度的影响。统计显著性将以 p < 0.05 为标准。

20.数据管理制度：

本研究数据采集主要由专人进行记录，保存于研究者处。

1. Affirmation of good faith:

Serious Disclaimer: I am a registered author of “The effect of resistance training combined with vibration training on the IGF-1/PI3K/AKT/FOXO3 signaling pathway in elderly patients with sarcopenia”, which is a collaborative research work of Zhou Haoyang et al. under the supervision of Liao Yuanpeng. Except for those already cited in the text, this thesis does not contain the results of any other individual or collective work that has been published or written. We will ensure that the operation strictly follows the experimental protocol and the authenticity of the data records. There is no conflict of interest among the participants of this study. Individuals and collectives who have made significant contributions will be clearly identified and acknowledged in the text. I am fully aware that the legal consequences of this statement are my responsibility.

2. Research topics:

Effects of resistance training combined with vibration training on IGF-1/PI3K/AKT/FOXO3 signaling pathway in elderly patients with sarcopenia

3. Research proposal version number:

V1.0

4. Sources of funding:

College funded plus self-funded

5. Research context:

Sarcopenia is a progressive disease associated with aging, characterized by decreased skeletal muscle mass, decreased muscle strength, and/or decreased physical mobility. According to the Asian Working Group on Sarcopenia (AWGS) guidelines, the prevalence of sarcopenia in Chinese suburban elderly ≥60 years of age was 9.3%, including 6.4% in men and 11.5% in women. Adverse outcomes such as accidental falls, upper and lower extremity fractures, physical disability, cognitive impairment, decreased quality of life and even death are associated with the progression of sarcopenia. The changes in elderly patients with sarcopenia are not only reflected in clinical outcomes and physiologic outcomes, such as age-related inflammation, metabolic abnormalities, and endocrine changes.

In older adults (≥ 60 years of age), sarcopenia is associated with reduced levels of anabolic hormones as indicated by reduced IGF-1. Forkhead-Box Protein O3 (FOXO3) is a downstream member of the IGF-1 signaling pathway that plays a dominant role in protein degradation and anabolic/catabolic protein homeostasis in the musculoskeletal system. Low levels of insulin-like growth factor 1 (IGF-1) lead to high activity of FOXO3 through regulation of the PI3K/AKT pathway. The target gene produced is the ubiquitin ligase Atrogin-1, which causes skeletal muscle atrophy.Among the many control genes in the musculoskeletal system, FOXO3 is critical because it is inactivated during muscle growth . Thus, inactivation of FOXO3 may be a potential target for the treatment of muscle atrophy. Therefore, measures should be taken to prevent and/or delay the development of sarcopenia.

Due to the lack of particularly effective medications for the treatment of sarcopenia, current research suggests that non-pharmacological treatments, particularly exercise training, remain the most promising treatment modality for significantly improving muscle function and physical health in elderly patients with sarcopenia. Currently, resistance training (RT) is one of the most commonly used exercise training for elderly patients with sarcopenia. However, RT is technically demanding for older adults and prone to muscle fatigue. These may reduce compliance, especially in frail older adults, and are difficult to implement universally in clinical settings. Vibration training (VT), a method of training in different body positions using vibration platforms with different vibration frequencies and amplitudes, is a prospective strategy for improving sarcopenia in older adults. In recent years, VT has been increasingly used to delay muscle function loss in elderly patients with sarcopenia. meta-analysis results showed that VT could change the decreasing trend of muscle mass, muscle strength, and physical fitness; meanwhile, several studies confirmed that VT has a positive effect on sarcopenia. Therefore, VT combined with RT may be a potential alternative training method to improve sarcopenia.

6. Purpose of the study:

To investigate the effects of resistance training combined with vibration training on the IGF-1/PI3K/AKT/FOXO3 axis in patients with sarcopenia. And to assess whether the two exercise combined treatment strategies have a significant effect on physiological changes in myasthenia gravis patients at the molecular level through a single-blind randomized controlled trial.7. Inclusion and exclusion criteria:

Inclusion criteria:

Inclusion criteria were older adults between the ages of 65 and 80 years with a diagnosis of sarcopenia according to the AWGS 2019 criteria, as well as the absence of serious medical conditions such as uncontrolled hypertension, hyperlipidemia, hyperglycemia, and diabetes mellitus and the absence of untreated medical conditions such as gallstones or renal stones, as well as infectious diseases, and the participants did not have a regular exercise regimen for more than 3 months.

Exclusion Criteria:

1) uncontrolled hypertension, hyperlipidemia, or diabetes mellitus; 2) untreated gallstones, kidney stones, or infectious diseases; 3) pathological bone diseases or chronic inflammatory diseases (e.g., rheumatoid arthritis); 4) any disorders that limit the ability to complete the required exercises in the study; 5) cardiovascular disease requiring a pacemaker or having a malignant tumor; 6) acute fractures or severe osteoarthritis; .7) current use of medications known to significantly affect muscle metabolism or hormonal balance, including but not limited to systemic corticosteroids, anabolic steroids, or hormone replacement therapy. Additionally, all participants’ current use of medications for cardiovascular or metabolic conditions (e.g., beta-blockers, statins, insulin sensitizers) will be documented at baseline. If imbalances between groups are identified, these variables will be considered as covariates in the statistical analysis; 8) current or recent (within the past 3 months) use of nutritional supplements that may affect muscle metabolism or sarcopenia progression, such as protein powders, amino acids, vitamin D, or calcium supplements.

8. Design plan, design pattern diagram with SPIRIT template

Parallel Design Randomized Controlled Experiments

9. Sample size estimation:

The sample size was calculated to be at least 73 individuals using GPower, V.3.1.9.7 software with an alpha of 0.05,power (1 - β) of 0.80 and considering a 20% attrition rate according to previous related studies. Therefore, we will recruit 96 older adults, 24 in each group.

10. Methods of random and hidden grouping:

Adult older adults who volunteer to participate in the study will be screened for eligibility and then randomized equally to RT, VT, RVT, or control group. The 96 participants will be sequentially coded based on their enrollment time using Excel software and then the formula "=RAND()" will be used to generate the corresponding random sequence. To facilitate the randomization process, the random numbers generated for each participant will be sorted and the modulus operation "=MOD()" will be applied to assign each participant to one of the four groups based on the residuals - 0 for the RT group, 1 for the VT group, 2 for the RVT group, and 3 is the control group. The randomized sequence generated through these steps will sort and group the 96 participants. Randomization will be performed by professional information technology personnel to ensure that the integrity of the randomization and blinding process is maintained by keeping the recruitment and grouping of participants unknown to the researchers.

11. Blinding

Since subjects could easily perceive the exercise performed, the study was blinded only to the measurers and evaluators.

12. Measurement indicators:

Main indexes: grip strength, step speed, IGF-1 concentration, PI3K/AKT signaling pathway, FOXO3 protein activity.

Secondary Indicators: Skeletal Muscle Mass, Stand-Up-Walk Timing Test, SF-36 Health Self-Test.

13. Definition of participant validity determination:

Criteria for exclusion and dropout: (1) those who were incorrectly included in the study because they did not meet the above inclusion criteria; (2) those who did not follow the protocol of intervention and examination as stipulated in the study; and (3) subjects who withdrew from the study on their own during the period of the study's conduct after enrollment and those who used interventions that were not relevant to the study or that had a serious impact were excluded.

14. Ethical considerations:

Registered and approved by the Ethics Committee of Chengdu Institute of Physical Education under Grant No. 2024 [66].

15. Recruitment of participants:

Subjects will be recruited through regular educational lectures at partner community centers, promotional materials at community centers or on social media, and partner referrals. Participants will be screened by bioelectrical impedance analysis, grip strength, and gait speed as defined by AWGS 2019. Participants who meet the criteria for frailty will be invited to participate in our study at the Sichuan Key Laboratory of Sports Medicine. All participants will be informed of the potential benefits and risks before signing a written informed consent form. Subjects can unconditionally withdraw from the study at any time.

16. Collection of general information on participants:

One week before the official experiment, the subjects' name, age, gender, height, weight, exercise frequency, and injury history were registered.

17. Baseline indicators and observational projects:

Same as the measurement indicator.

18. Standard Operating Procedures:

(1) Experimental training

The RT, VT and RVT groups were pre-trained to familiarize them with the appropriate training sessions. Formal training will be conducted after the pre-training.The 3 intervention groups (RT group, VT group and RVT group) will undergo a 5 min warm-up and cool-down training session before and after the intervention. All training sessions will be conducted by a physical therapist. Throughout the training period, the researcher will record the participants' attendance and compliance. If for some reason a subject is unable to attend the training on time, the training will be conducted at another time during that week. All subjects will not be allowed to perform additional physical activities and/or change their daily routine during the experimental period.

(2) Resistance training group

Participants assigned to the RT group will undergo a 12-week exercise program comprising 3 weekly sessions. Before the intervention begins, all participants will perform a one-repetition maximum (1RM) test to elucidate baseline muscle strength and determine individualized training intensities. The 1RM will be estimated using a submaximal testing approach, in which participants perform 3–5 repetitions at approximately 60%–80% of their perceived maximum load for selected TheraBand-based resistance exercises (e.g., knee extension, hip abduction, elbow flexion). The Brzycki formula (estimated 1RM (kg) = submaximal weight (kg) / (1.0278 − 0.0278 × maximal number of repetitions)) will be applied to estimate 1RM values from these submaximal repetitions. All tests will be supervised by experienced physiotherapists, and standardized rest intervals of 1 minute will be maintained between sets to minimize fatigue. Based on the estimated 1RM, participants will be assigned an appropriate TheraBand (color-coded by resistance level: yellow, red, green, blue, black) and instructed to maintain a specific elongation length (e.g., 100% or 200%) to approximate the desired resistance. This allows adjustment of load to approximately 70% of 1RM. To enhance precision, perceived exertion will be concurrently monitored using the Borg CR-10 scale, ensuring training load consistency throughout the intervention. The training intensity will be set at 70% of the 1RM for the entire 12-week program. Each training session will comprise 3 phases: a 5-minute warm-up, the main 30-minute RT exercises, and a 5-minute cool-down. During the 5-minute warm-up, participants will mainly perform neck, shoulder, lower back, hip, knee, and ankle stretches. Participants will perform three sets of 10 repetitions for each exercise, with a 1-minute rest between sets. The exercises will be performed using a TheraBand to reduce the stress of free weights on participants’ joints and increase their motivation to engage in RT. RT exercises will focus on lower limb muscles (knee flexion/extension, hip abduction), upper limb muscles (shoulder extension/abduction, elbow flexion/extension), and core muscles (including chest presschest depression). Following these exercises, participants will perform a 5-minute cool-down protocol matching the warm-up in structure..

(3) Vibration Training Group

The VT group received vibration training (VT) for 20 min 3 times per week for 12 weeks. Vibration Training (VT) was performed using a vibration platform (Pro5 AIRdaptive, Power Plate, USA) that generates vertical vibration. Each session lasted approximately 20 minutes and consisted of a 5-minute warm-up, 10 minutes of vibration and a 5-minute cool-down. The frequency of vibration was gradually increased to 40 Hz with a peak amplitude of 4 mm. During VT, subjects will be asked to stand barefoot on the vibration platform with their knees flexed at 60° and, if required, hold the vibrating cord with both hands for balance. The VT exercise should be held for 1.5 minutes, and repeated for 4 sets per day with a 1-minute rest in between each set.

(4) Resistance training combined with vibration training group

The Resistance Vibration Training group (RVT group) will receive 12 weeks of resistance vibration training three times per week. At the end of each resistance training session, additional vibration training will be performed with the same setup parameters as the VT group.

(5) Control group

Control subjects received two 60-minute educational sessions per month at a community center. The educational sessions were taught by invited experts on the topic of aging, health and sarcopenia, including the definition of sarcopenia, its causes, pathology, clinical manifestations and adverse consequences for the elderly.

19. Methods of statistical analysis:

IBM SPSS v.20.0 will be utilized to analyze all the data. The primary outcomes with Holm–Bonferroni pairwise comparisons, will be assessed via two-way repeated-measures analysis of variance (ANOVA; time points: baseline, 12, 24 weeks; groups: control, RT, VT, RVT). Missing data will be imputed via multiple imputation, and covariates (baseline IGF-1, age, sex, muscle mass) will be included in the analysis of covariance models. Effect sizes (partial η2 for ANOVA; for pairwise comparisons, the Cohen’s d with 95% confidence intervals were employed) will be categorized as small (η2 ≥ 0.01, d ≥ 0.2), medium (η2 ≥ 0.06, d ≥ 0.5), or large (η2 ≥ 0.14, d ≥ 0.8), accompanied by exact p-values. Longitudinal secondary outcomes (e.g., gait speed) will be analyzed via linear mixed models, reporting time-by-group interaction p-values. Exploratory subgroup analyses (p < 0.10) will be used to examine sex and baseline severity effects. Statistical significance will be considered at a p < 0.05.

20. Data management systems:

Data collection for this study was mainly recorded by a person and kept with the researcher.
