# Supplementary material for: Impacts of resistance training combined with vibration training on the IGF-1/PI3K/AKT/FOXO3 axis and clinical outcomes in patients with sarcopenia: A protocol for a randomized controlled trial
Source: PLoS One. 2025 Sep 26;20(9):e0333343. doi: 10.1371/journal.pone.0333343 (PMC12468885; doi:10.1371/journal.pone.0333343)
Supplement: Attachment — Submitted filename: Response to Reviewers.docx [file pone.0333343.s005.docx]

Dear Editor and Reviewers,

We sincerely appreciate the time and effort that you and the reviewers have devoted to evaluating our manuscript entitled **“Impacts of resistance training combined with vibration training on the IGF-1/PI3K/AKT/FOXO3 axis and clinical outcomes in patients with sarcopenia: A protocol for a randomized controlled trial”** (Manuscript ID: [PONE-D-25-28564]). We are grateful for the constructive comments and insightful suggestions, which have greatly helped us improve the quality and clarity of the manuscript.

In response to each comment, we have carefully revised the manuscript and provided point-by-point responses below. We hope that the revised version meets the journal’s requirements and addresses all concerns raised during the review process.

**Reviewer #1:** Overall Assessment:

This research protocol aim to investigate the effects of resistance training, vibration training, and their combination on sarcopenia, with a focus on both clinical outcomes and the intracellular IGF-1/PI3K/AKT/FOXO3 signaling axis, addresses a critical gap in the literature. The paucity of human-based evidence concerning these specific molecular pathways in the context of sarcopenia interventions significantly elevates its clinical and academic merit.

Specific Points of Review and Recommendations for Improvement:

1. Methodological Concerns Regarding Molecular Mechanism Elucidation:

The proposed timing of biopsy sample collection appears to be a limiting factor in fully elucidating the intricate molecular mechanisms underpinning the observed clinical effects. Without a more comprehensive sampling strategy (e.g., acute post-intervention time points or multiple time points throughout the intervention period), the study's capacity to precisely delineate the temporal molecular adaptations within the IGF-1/PI3K/AKT/FOXO3 axis remains ambiguous. A more detailed justification for the chosen biopsy time points, or a consideration of additional sampling, is warranted to strengthen the mechanistic insights.

Reply: We sincerely thank the reviewer for this important comment. To clarify, our current protocol includes muscle biopsies at three time points: baseline (pre-intervention), 12 weeks (post-intervention), and 24 weeks (follow-up). These time points were selected to observe cumulative molecular adaptations before, after, and during the sustained effects following the intervention. We fully agree that more frequent sampling (e.g., acute post-exercise or weekly biopsies) could offer deeper insights into the transient and temporal dynamics of the IGF-1/PI3K/AKT/FOXO3 axis. However, given the invasive nature of muscle biopsy and ethical considerations involving older participants with sarcopenia, we opted to minimize the participant burden and risk by limiting the number of biopsies. In future studies, we plan to explore less invasive approaches such as circulating biomarkers or real-time monitoring techniques, or use animal models to assess rapid molecular responses during intervention. We have added a clarification in the Methods section to explain our rationale and acknowledge this limitation.

2. Introduction:

Sex-Specific Considerations: The introduction lacks sufficient discussion regarding the known sex differences in sarcopenia prevalence, progression, and responsiveness to exercise interventions. Given the potential for differential responses, it is imperative to integrate relevant literature on sex-specific aspects and outline how these considerations will be addressed within the study design and subsequent analyses. This will enhance the generalizability and clinical relevance of the findings.

Reply: We appreciate the reviewer’s thoughtful suggestion regarding the inclusion of sex-specific considerations. We fully agree that sex differences in the sarcopenia prevalence, pathophysiology, and responsiveness to exercise are well-documented and clinically important. To address this, we revised the Introduction section to include additional reference citations on sex-specific patterns of sarcopenia. For instance, studies have shown that women may experience a more rapid decline in muscle strength relative to muscle mass whereas men tend to exhibit more substantial losses in skeletal muscle mass with age. Moreover, differences in hormonal status, fiber-type composition, and neuromuscular control may influence exercise responsiveness according to sex. In the “Statistical analysis” section, we have clarified that sex will be included as a covariate in the ANCOVA and linear mixed models, and exploratory subgroup analyses by sex will also be conducted to identify potential differential effects. We believe that these additions enhance the clinical relevance and generalizability of the study findings.

Elaboration on Vibration Training (VT) Molecular Mechanisms: The hypothesized molecular mechanisms by which vibration training impacts the IGF-1/PI3K/AKT/FOXO3 axis require more thorough substantiation. While general benefits of VT are often cited, a more detailed review of existing pre-clinical (e.g., animal models) and preliminary human studies that explore the cellular and molecular effects of VT on muscle anabolism and catabolism is necessary. This includes explicitly stating the proposed molecular cascade and formulating clear, testable hypotheses regarding VT's specific influence on the IGF-1/PI3K/AKT/FOXO3 axis.

Reply: We thank the reviewer for this excellent comment. We agree that a more detailed explanation of the hypothesized molecular mechanisms of vibration training (VT) is essential for enhancing the theoretical foundation of the study. To address this, we have expanded the Introduction section to include additional evidence from both animal models and preliminary human studies. These studies suggest that VT may stimulate muscle anabolism via the upregulation of IGF-1 expression and downstream activation of the PI3K/AKT signaling cascade, leading to inhibition of FOXO3 nuclear translocation and suppression of proteolytic genes such as MuRF-1 and Atrogin-1. Furthermore, we have clarified the proposed mechanism in our study, as follows. VT may enhance mechanical loading via muscle spindle stimulation and neuromuscular activation, thereby activating mechanosensitive signaling pathways that converge on IGF-1 synthesis. This upregulation is hypothesized to activate PI3K/AKT and suppress FOXO3-mediated muscle degradation. We have now explicitly stated this hypothesis in the revised manuscript and believe it will improve the mechanistic clarity of the protocol.

2. Research Methods:

Design and Grouping – Justification for Intervention and Follow-up Durations: The rationale for the "12-week intervention" and "24-week follow-up" periods is not adequately articulated. A robust justification, grounded in existing literature on exercise physiology, sarcopenia intervention guidelines, or pilot data, is essential to validate these specific durations. This should include considerations for achieving meaningful physiological adaptations and assessing the sustainability of effects.

Reply: We appreciate the reviewer’s thoughtful comment. The selection of a 12-week intervention period was based on extensive literature on exercise physiology and sarcopenia interventions. Previous studies have demonstrated that 12 weeks of resistance or vibration training is sufficient to induce measurable improvements in muscle strength, mass, and hormonal signaling, including IGF-1 pathway activation (Kan et al. 1989; Pereira et al. 2024; Tieland et al. 2012). Specifically, a 12-week duration allows for both neural and hypertrophic adaptations to occur, while maintaining participant compliance in older adults. The 24-week follow-up (i.e., 12 weeks post-intervention) was included to evaluate the sustainability of physiological and molecular adaptations after training cessation. This time frame has been adopted in prior studies on sarcopenia to assess whether benefits in strength and function persist, decline, or plateau in the absence of continued training (Chittrakul et al. 2020). We have now elaborated on these rationales in the “Study design and grouping” section of the revised manuscript.

Sample Size Determination – Consideration of Sex: The current sample size calculation appears to lack consideration for potential sex-specific differences in outcome measures and intervention effects. If significant sex differences are anticipated in sarcopenia progression or response to interventions, the sample size calculation should either account for these variations or explicitly state the implications for generalizability across sexes. Stratification by sex or a power analysis informed by sex-specific variability would enhance the methodological rigor.

Reply: We thank the reviewer for this thoughtful comment. We have revised the “Statistical analysis” section to clarify that sex, along with other key covariates, will be adjusted for using ANCOVA models. Additionally, we will conduct exploratory subgroup analyses stratified by sex and examine sex-by-intervention interaction terms to evaluate potential moderation effects. Although the current sample size is not specifically powered for interaction effects, these analyses will help identify sex-specific trends and guide future trials. This information has been added to the revised manuscript (Methods > Statistical analysis)

Inclusion Criteria – Management of Medication History: The inclusion criteria require more stringent attention to participants' medication history. Pharmacological agents known to influence muscle metabolism, inflammation, or hormonal balance (e.g., corticosteroids, specific cardiovascular medications, diabetes medications, etc.) could confound the study outcomes. A clear strategy for managing these potential confounding factors, whether through explicit exclusion criteria, precise documentation, or statistical adjustment in the analysis, must be elaborated.

Reply: We appreciate the reviewer’s valuable recommendation. In response, we have revised the “Exclusion criteria” section to explicitly exclude participants who are currently receiving medications that are known to substantially affect muscle metabolism or hormonal regulation, including systemic corticosteroids, anabolic agents, or hormone replacement therapy. Additionally, the use of medications for cardiovascular or metabolic conditions (e.g., beta-blockers, statins, insulin sensitizers) will be carefully documented during screening and baseline assessment. These variables will be considered covariates in the statistical analysis if they show imbalance between groups or correlations with outcome measures.

**Reviewer #2:** The primary objective of this 2x2 factorial clinical trial is to evaluate the potential in vivo modulation of molecular mechanisms through combined resistance training (RT) and vibration training (VT) in patients with sarcopenia, and to determine the intervention’s impact on physical function. A total of 96 patients will be randomly allocated to one of four treatment arms: control, RT, VT, and RVT (combined RT and VT), with 24 patients per arm. The intervention period will last 12 weeks, and assessments will be conducted at baseline, after 12 weeks (post-intervention), and at 24 weeks (follow-up). Primary outcome measures include gait speed, handgrip strength, and skeletal muscle mass. Secondary outcome measures encompass IGF-1 concentrations, PI3K/AKT and FOXO3 protein activities, quality of life, and timed-up-and-go test performance. Although the authors discussed the statistical considerations, including the power analysis and sample size estimation, there are several major statistical concerns about this protocol.

Statistical critiques:

1. Although the authors provided a section on sample size determination, the information presented is superficial. The authors should explicitly clarify: (1) the specific statistical tests used for determining the sample size, (2) the anticipated effect sizes among the four treatment arms for each of the three primary endpoints, along with clinical justifications, (3) the identification of the primary measurement time point(s), considering assessments at both 12 and 24 weeks, (4) adjustment methods for the type I error rate, given the multiple comparisons arising from four treatment arms (six possible pairwise comparisons), three primary endpoints, and two assessment time points, and (5) whether an additive or synergistic effect between RT and VT is anticipated, as this significantly influences the power analysis and required sample size.

Reply: We thank the reviewer for this detailed and insightful comment. We have revised the “Sample size determination” section to address each of the concerns listed below.

(1) The sample size was calculated based on two-way repeated-measures ANOVA with interaction, using G*Power 3.1.

(2) Anticipated effect sizes (partial η^2^ = 0.14, corresponding to Cohen's f = 0.40) were derived from previous studies examining the effects of resistance and vibration training on IGF-1 expression and muscle function in older adults. These represent moderate-to-large clinically meaningful changes.

(3) The primary endpoint is the change from baseline to 12 weeks, which aligns with the end of the supervised intervention and is expected to capture peak molecular and functional adaptations. The 24-week point serves as a follow-up to assess sustainability but was not used in powering the study.

(4) Although multiple endpoints and comparisons exist, we acknowledge the inflation of type I error. Therefore, Holm–Bonferroni correction will be applied in the main analysis; this has been reflected in the power calculation assumptions.

(5) We hypothesize an additive effect of RT and VT, rather than synergistic interaction. Thus, no interaction effect was assumed in the initial power analysis. Future analyses will explore potential synergy via interaction terms, but the current sample size ensures detection of additive group differences.

The revised paragraph is now included in the “Sample size determination” section of the Methods.

2. The authors need to clearly state the randomization method employed (e.g., simple randomization, permuted block randomization). Additionally, they should specify if any baseline variables will be used as stratification factors during randomization.

Reply: We appreciate the reviewer’s important observation. We have clarified the randomization method in the “Randomization and blinding” section. Specifically, a permuted block randomization (block size of 4) will be used to ensure balanced group sizes. Randomization will be conducted by an independent statistician using a computer-generated random sequence. No stratification by baseline variables (e.g., age, sex) will be used in the randomization process. However, these variables will be included as covariates in the statistical analysis to control for potential confounding.

3. It is unclear why the authors did not propose using mixed-effects models for analyzing both primary and secondary outcomes. The authors should clarify their rationale for choosing alternative methods or consider adopting mixed-effects models to accommodate repeated measures and potential within-subject correlations.

Reply: Thank you for your suggestion. In the revised manuscript, we clarified that two-way repeated-measures ANOVA will be used for primary outcomes owing to the balanced design. However, we have now specified the use of linear mixed-effects models for secondary longitudinal outcomes (e.g., gait speed), which can better accommodate within-subject correlations and missing data.

4. The authors should explicitly specify the statistical methods intended for calculating 95% confidence intervals (CIs) for all primary and secondary endpoints.

Reply: We appreciate the reviewer’s valuable comment. In the revised manuscript, we have now explicitly stated that 95% confidence intervals for all effect sizes (Cohen’s d) and model estimates (e.g., from linear mixed models) will be computed using bias-corrected and accelerated (BC) bootstrap methods or model-based standard errors, depending on the test applied.

5. The authors should state clearly whether the data analysis will follow an intention-to-treat approach or a modified intention-to-treat methodology.

Reply: We thank the reviewer for this important suggestion. In the revised manuscript, we have clarified that data analysis will be conducted based on the intention-to-treat (ITT) principle. All randomized participants will be included in the analysis according to their originally assigned groups, regardless of adherence or withdrawal. This approach will help to preserve the benefits of randomization and ensure the robustness of the study findings.

6. The authors should clearly outline their strategy for multivariable data analysis, addressing the following aspects: (1) model performance evaluation, (2) assumption verification, (3) missing data handling, (4) adjustments for interaction terms and potential nonlinear relationships, and (5) methods planned for internal validation of the analytical model.

Reply: We thank the reviewer for raising this important point. The revised “Statistical analysis” section now provides a more detailed description of our multivariable modeling approach. Specifically, model performance will be evaluated via effect sizes (partial η^2^, Cohen’s d) and model-based estimates with 95% confidence intervals. For non-normally distributed estimates, we will adopt the bias-corrected and accelerated (BCa) bootstrap method (1,000 resamples). Normality and variance assumptions will be verified via Q–Q plots, Shapiro–Wilk, and Levene’s tests. Missing data will be handled using multiple imputation assuming missing-at-random (MAR), with sensitivity analyses to assess robustness. We will test predefined interaction terms (e.g., sex × group × time), and exploratory nonlinear patterns may be explored using generalized additive models, if indicated. Internal model stability and confidence will be evaluated using bootstrap resampling (n = 1,000), particularly for subgroup and sensitivity analyses. All analyses will follow the intention-to-treat (ITT) principle.

7. It is unclear whether the authors have considered including an interim analysis plan.

Reply: We appreciate the reviewer’s inquiry. Given that this is a single-center, relatively short-term (24-week) randomized controlled trial with a moderate sample size (n = 96), we have not planned for a formal interim analysis. The study duration and sample size were determined with the aim to minimize risk exposure and are consistent with prior sarcopenia intervention trials of similar scale. Moreover, the intervention modalities (RT, VT, RVT) are low-risk and have been extensively studied in older populations, further reducing the need for interim efficacy or safety assessments. However, we will conduct routine safety monitoring throughout the trial; any adverse events will be promptly documented and reported to the institutional ethics committee.

Reviewer #3: Preventing sarcopenia is a serious worldwide social issue. Resistance exercises are an evidence-based solution to this problem. If there is a way to increase the effectiveness of resistance exercises using a safe and/or low-cost manner, it should be challenged. The proposal in this article, written by Zhou H et al., is interesting from this point of view. However, content revisions and additional information are needed to make this proposal more understandable to the readers.

1) In community-dwelling elderly, the first screening to detect sarcopenia has been effective and recommended for physical function and muscle strength tests. However, loss of muscle mass is the most important indicator in the diagnosis. Therefore, muscle mass should be described before other indicators when describing sarcopenia in an academic article. For example, lines 26-27 (page 3), lines 39-40 (page 3), and line 49 (page 4) should bring the words “size” or “muscle mass” before the others.

Reply: We thank the reviewer for this valuable suggestion. We fully agree that muscle mass loss constitutes the most critical and specific criterion in the diagnosis of sarcopenia, as recognized by consensus definitions (e.g., EWGSOP2 and AWGS 2019). Accordingly, we have revised the Introduction and Methods sections to describe muscle mass before muscle strength and physical function, ensuring appropriate emphasis on this primary diagnostic component.

2) Lines 50-52: The word “in China” should be included in the text because it is difficult to understand whether the 6.4% in men and 11.5% in women with sarcopenia are in China.

Reply: Thank you for your helpful suggestion. We agree that the prevalence values should clearly indicate the geographic context. We have revised the relevant text in the Introduction.

3) Lines 87-88: Correct the sentence as this sentence does not mean “delay the muscle function” but “delay the decline in muscle function”.

Reply: Thank you for pointing this out. We have revised the sentence accordingly, to “delay the decline in muscle function,” so as to improve clarity and accuracy.

4) Lines 114 and 119: “Fig” at the beginning of a sentence should be written as “Figure”.

Reply: Thank you for your suggestion. We have made the corresponding revisions in the manuscript.

5) Line 183: Explain in detail the procedures and equipment that the authors plan to use to measure 1 RM.

Reply: Thank you for your thoughtful comment. We have added detailed procedures and equipment descriptions for estimating one-repetition maximum (1RM). Specifically, a submaximal testing approach will be used in which participants perform 3–5 repetitions at 60%–80% of their perceived maximal effort for selected TheraBand-based resistance exercises (e.g., knee extension, hip abduction, elbow flexion). The Brzycki formula (estimated 1RM (kg) = submaximal weight (kg) / (1.0278 − 0.0278 × maximal number of repetitions)) (Brzycki 1993) will then be applied to estimate 1RM values. All tests will be supervised by experienced physiotherapists, and standardized rest intervals will be maintained to minimize fatigue. This method has been widely adopted in older adult populations to ensure safety and feasibility. The revised content has been incorporated into the Methods section.

6) Line 184 and S3 file: The authors plan to use TheraBands in training sessions, but it is unclear how to adjust the training load to 60% or 70% of 1 RM. Explain in detail how to adjust the load so that readers can understand.

Reply: Thank you for your insightful comment. We have now added a detailed description of how resistance loads were calibrated using TheraBands to approximate percentages of 1RM. Specifically, a submaximal testing approach was used to estimate each participant’s 1RM; based on these values, appropriate TheraBand resistance levels (color-coded) and elongation lengths (e.g., 100%–200%) were selected. This method is supported by previous literature on elastic band resistance training (Lu et al. 2022; Zhang et al. 2024). Additionally, perceived exertion is concurrently monitored using the Borg CR-10 scale (Borg 1982; Oliveros R et al. 2011) to ensure training intensity remains within the target ranges. These revisions have been made in the Methods section to improve transparency and replicability.

7) Line 193: The term “chest depression” is not commonly used in training science. The term should be replaced with “chest press”.

Reply: Revised. Thank you.

8) Lines 202-203: If the VT consists of 4 sets of 1.5 minutes per session, with 1 minute of interval between the sets, the authors describe the total as 10 minutes (line 199), but isn’t 9 minutes the correct total?

Reply: Thank you for your careful observation. We agree with the reviewer that the total duration should be 9 minutes, not 10 minutes, considering four sets of 1.5 minutes with three 1-minute intervals. The manuscript has been corrected accordingly.

9) Lines 184-185, 207-208: Why is it planned for the RT (70% of the 1 RM for the entire 12-week program) and RVT (50% of 1RM for the first 4 weeks, 55% of 1 RM for the second 4 weeks, and 60% of 1 RM for the final 4 weeks) groups to undergo resistance training at different exercise intensities? Given that the RVT group will be subjected to a lower intensity, any observed difference in training outcomes between the two groups may be confounded by this discrepancy, making it difficult to conclude whether the effects are attributable to training intensity or vibration stimulation. Would it not be more appropriate for both groups to be trained at the same resistance exercise intensity to ensure comparability? If differing training intensities are planned to be employed, the authors should explain scientifically the approach.

Reply: Thank you for your valuable comment. We acknowledge that different training intensities between the RT and RVT groups may raise concerns regarding potential confounding effects. The gradual progression in the RVT group (from 50% to 60% of 1RM) was deliberately designed to ensure safety and improve adherence, particularly given the novelty of combining vibration stimulation with resistance training in older adults. This phased approach is consistent with prior studies involving vibration-enhanced resistance training in aging populations (Bogaerts et al. 2007; Machado et al. 2010), which emphasizes a need for initial adaptation to vibration exposure to reduce the risk of musculoskeletal discomfort or dropout. In contrast, the RT group—without the added neuromuscular stimulus of vibration—was assigned a constant moderate-to-high intensity (70% 1RM) based on established recommendations for sarcopenia intervention. We have revised the manuscript to clarify this rationale.

10) Lines 214-217: Why do the authors allow the control group to take educational courses? Education can cause behavioral changes in the control group, which may lead to lifestyle changes. Should the RT and RVT groups also take the educational courses? Add a rationalization that no matter what the research results of the study, having only the control group take the educational courses will not interfere with the interpretation of the research results.

Reply: Thank you for raising this important methodological point. Weekly health education sessions provided to the control group are solely intended to ensure participant engagement and minimize attrition. The content is general, non-individualized, and not designed to promote physical activity or muscular enhancement. These sessions are not expected to produce physiological effects related to the study’s primary endpoints. Therefore, we believe that the inclusion of education only in the control group will not interfere with the interpretation of intervention effects. A clarification of this point has been added to the manuscript.

11) Line 225: Revise “have sarcopenia” to “have probable sarcopenia” because decreased handgrip strength alone is not sarcopenia, but probable sarcopenia.

Reply: Thank you for pointing this out. We have revised the relevant context.

12) Lines 220, 228-231: Since the primary outcomes in the study include not only strength and performance (line 220) but also muscle mass (lines 228-231), the content of lines 228-231 should be included at the beginning (line 220).

Reply: Thank you for pointing this out. We have revised the relevant paragraph.

13) Nutritional intake is also important in sarcopenia, but do the authors not plan to survey it? The authors should explain why the interpretation of the results would not be impaired if they are not done.

Reply: We appreciate the reviewer’s important observation regarding the role of nutrition in sarcopenia-related outcomes. Whereas our study did not incorporate formal dietary assessments, we acknowledge that recent changes in nutritional supplementation may influence muscle metabolism and thereby confound the intervention effects. To address this concern, we have revised the exclusion criteria to explicitly exclude participants who have received nutritional supplementation (e.g., protein powders, amino acids, or vitamin D/calcium supplements) in the previous 3 months. This is aimed at minimizing confounding and improving internal validity. We have also added a note in the manuscript to acknowledge the lack of formal dietary monitoring as a study limitation.

14) Revise “Gait speed (>1 m/s)” in Figure 2 to “Gait speed (<1 m/s)”.

Reply: Thank you for pointing this out. We have revised the figure.

15) S3 file, lines 2-3, English page 6: The following sentence is difficult to understand the meaning, “Each movement will be repeated 10 times, with 3 repetitions per set…”. Does “3 repetitions per set” in the sentence mean “3 sets per session”?

Reply: Thank you for your helpful comment. The sentence, “Each movement will be repeated 10 times, with three repetitions per set” was indeed unclear and incorrectly phrased. We have revised it as follows: “Each movement will be performed in three sets of 10 repetitions, with a 1-minute rest between sets,” which more accurately reflects the intended training protocol.

Bogaerts A, Verschueren S, Delecluse C, Claessens AL, and Boonen S. 2007. Effects of whole body vibration training on postural control in older individuals: a 1 year randomized controlled trial. *Gait & Posture* 26:309-316.

Borg GA. 1982. Psychophysical bases of perceived exertion. *Medicine and Science In Sports and Exercise* 14:377-381.

Brzycki M. 1993. Strength Testing—Predicting a One-Rep Max from Reps-to-Fatigue. *Journal of Physical Education, Recreation & Dance* 64:88-90. 10.1080/07303084.1993.10606684

Chittrakul J, Siviroj P, Sungkarat S, and Sapbamrer R. 2020. Multi-System Physical Exercise Intervention for Fall Prevention and Quality of Life in Pre-Frail Older Adults: A Randomized Controlled Trial. *International Journal of Environmental Research and Public Health* 17. 10.3390/ijerph17093102

Kan N, Okino T, Nakanishi M, Sato K, Mise K, Yamasaki S, Teramura Y, Ohgaki K, and Tobe T. 1989. Therapeutic efficacy of sequential therapy with OK-432, cyclophosphamide, IL2-cultured lymphocytes and in vivo IL2 against advanced murine plasmacytoma. *Biotherapy (Dordrecht, Netherlands)* 1:197-206.

Lu L, He X, Ma L, Liu Y, and Chen N. 2022. Effects of vibration training vs. conventional resistance training among community-dwelling older people with sarcopenia: three-arm randomized controlled trial protocol. *Frontiers In Aging Neuroscience* 14:905460. 10.3389/fnagi.2022.905460

Machado A, García-López D, González-Gallego J, and Garatachea N. 2010. Whole-body vibration training increases muscle strength and mass in older women: a randomized-controlled trial. *Scandinavian Journal of Medicine & Science In Sports* 20:200-207. 10.1111/j.1600-0838.2009.00919.x

Oliveros R MS, Avendaño M, Bunout D, Hirsch S, De La Maza MP, Pedreros C, and Müller H. 2011. [A pilot study on physical training of patients in hemodialysis]. *Revista Medica de Chile* 139:1046-1053.

Pereira B, Monteiro D, Matos R, Jacinto M, Amaro N, Antunes R, and Rodrigues F. 2024. Effect of a 12-Week Strength Training Program on Muscle Strength Measures of Institutionalized Older Adults-A Pilot Study. *Healthcare (Basel, Switzerland)* 12. 10.3390/healthcare12141428

Tieland M, Dirks ML, van der Zwaluw N, Verdijk LB, van de Rest O, de Groot LCPGM, and van Loon LJC. 2012. Protein supplementation increases muscle mass gain during prolonged resistance-type exercise training in frail elderly people: a randomized, double-blind, placebo-controlled trial. *Journal of the American Medical Directors Association* 13:713-719. 10.1016/j.jamda.2012.05.020

Zhang M, Song Y, Zhu J, Ding P, and Chen N. 2024. Effectiveness of low-load resistance training with blood flow restriction vs. conventional high-intensity resistance training in older people diagnosed with sarcopenia: a randomized controlled trial. *Scientific Reports* 14:28427. 10.1038/s41598-024-79506-9
