# Supplementary material for: Impacts of resistance training combined with vibration training on the IGF-1/PI3K/AKT/FOXO3 axis and clinical outcomes in patients with sarcopenia: A protocol for a randomized controlled trial
Source: PLoS One. 2025 Sep 26;20(9):e0333343. doi: 10.1371/journal.pone.0333343 (PMC12468885; doi:10.1371/journal.pone.0333343)
Supplement: Attachment — Submitted filename: Response_to_Reviewers_auresp_2.docx [file pone.0333343.s006.docx]

Dear Editor and Reviewers,

We sincerely thank you for your time, effort, and constructive feedback on our manuscript entitled “Impacts of resistance training combined with vibration training on the IGF-1/PI3K/AKT/FOXO3 axis and clinical outcomes in patients with sarcopenia: A protocol for a randomized controlled trial” (Manuscript ID: PONE-D-25-28564R1).

We are grateful for the valuable comments and suggestions provided in both the first and second rounds of review, which have greatly helped us improve the clarity, rigor, and overall quality of our work. We are particularly encouraged by the reviewers’ recognition that the revised manuscript has been substantially improved.

In this revised version, we have carefully addressed all the remaining concerns raised by the reviewers. Specifically, we further clarified the rationale and potential influence of the educational sessions in the control group, and we added relevant references to support our explanation. All changes have been highlighted in the revised manuscript for ease of review.

We once again thank the reviewers and the editor for their insightful comments and guidance, which have significantly strengthened our manuscript. We hope that our revisions meet with your approval.

Reviewer #1: The authors have successfully addressed all of my previous comments. The manuscript is much improved, and I have no further concerns at this time.

Response to Reviewer #1: We sincerely appreciate your positive feedback and recognition of our revisions. We are very grateful for your constructive comments in the first round, which greatly helped us improve the quality of the manuscript. Thank you again for your time and support.

Reviewer #2: The manuscript is much improved.

Response to Reviewer #2: We sincerely thank you for your positive evaluation of our revised manuscript. We greatly appreciate your comments and recognition, which encouraged us to further refine and improve our work. Thank you again for your valuable time and support.

Reviewer #3: The revised manuscript by Zhou H et al. almost responded to my questions. However, there is one point that needs to be revised.

1) The authors revised that the educational sessions for the control group will not influence behaviors related to daily physical activity levels or nutritional intake in elderly people (lines 312 to 318). Many studies have reported that posters encouraging people of various ages to quit smoking are effective in promoting smoking cessation, and that posters encouraging exercise increase the physical activity levels people engage in. In this way, it has been shown that providing even a small amount of information can lead to behavioral change. Thus, it is difficult to interpret that the educational sessions for the elderly people, which consisted of two 60-minute classes per month on sarcopenia and health, will not promote behavioral change in elderly people. Previous studies showing that such educational interventions do not promote behavioral change, or even if they do, they do not result in improved muscle strength or physical function, should be presented. It is important to carefully evaluate the effects of educational sessions, as otherwise there is a risk of underestimating the value of physical training in other groups.

Response to Reviewer #3: We sincerely thank you for this important comment. We fully agree with your concern that educational interventions may induce behavioral changes in elderly people, as suggested by previous evidence from public health campaigns. To address this, we have carefully revised the manuscript:

In the Methods – Educational courses section, we clarified that the educational sessions were designed as general health information lectures, standardized and non-interactive, with no specific guidance on exercise practice or nutrition planning.

We have now added references to previous studies reporting that educational lectures alone did not lead to significant improvements in muscle strength, mass, or functional outcomes in elderly populations with sarcopenia or frailty.

In the Discussion section, we further acknowledged that although minor behavioral changes cannot be excluded, prior evidence indicates that education-only interventions rarely translate into measurable improvements in physical performance. Therefore, the risk of overestimating the effects in the control group is minimal.

We sincerely appreciate your suggestion, which helped us refine the rationale of our control group design and avoid potential underestimation of the exercise interventions.
